# Long-Term Anticoagulant Therapy of Patients with Venous Thromboembolism. What Are the Practices?

**DOI:** 10.1371/journal.pone.0128741

**Published:** 2015-06-15

**Authors:** Isabelle Mahé, Raluca Sterpu, Laurent Bertoletti, Luciano López-Jiménez, Meritxell Mellado Joan, Javier Trujillo-Santos, Aitor Ballaz, Luis Manuel Hernández Blasco, Pablo Javier Marchena, Manuel Monreal, RIETE investigators

**Affiliations:** 1 Department of Internal Medicine, Hôpital Louis Mourier, Colombes (APHP), University Paris 7, EA REMES 7334, Université Paris Diderot, Sorbonne Paris Cité, France; 2 Department of Internal Medicine, Hôpital Louis Mourier, Colombes, (APHP), University Paris 7, Paris, France; 3 Department of Vascular and Therapeutic Medicine, CHU Saint-Etienne, Hôpital Nord, Saint-Etienne, France; 4 Department of Internal Medicine, Hospital Provincial Reina Sofía, Córdoba, Spain; 5 Department of Angiology and Vascular Surgery, Hospital del Mar, Barcelona, Spain; 6 Department of Internal Medicine, Complejo Hospitalario Universitario de Cartagena, Murcia, Spain; 7 Department of Pneumonology, Hospital de Galdakao, Vizcaya, Spain; 8 Department of Pneumonology, Hospital General Universitario de Alicante, Alicante, Spain; 9 Department of Internal Medicine, Parc Sanitari Sant Joan de Deu-Hospital General, Barcelona, Spain; 10 Department of Internal Medicine, Hospital Universitari Germans Trias i Pujol, Badalona, Spain; Ohio State University, UNITED STATES

## Abstract

Current guidelines of antithrombotic therapy suggest early initiation of vitamin K antagonists (VKA) in non-cancer patients with venous thromboembolism (VTE), and long-term therapy with low-molecular weight heparin (LMWH) for those with cancer. We used data from RIETE (international registry of patients with VTE) to report the use of long-term anticoagulant therapy over time and to identify predictors of anticoagulant choice (regarding international guidelines) in patients with- and without cancer. Among 35,280 patients without cancer, 82% received long-term VKA (but 17% started after the first week). Among 4,378 patients with cancer, 66% received long term LMWH as monotherapy. In patients without cancer, recent bleeding (odds ratio [OR] 2.70, 95% CI 2.26–3.23), age >70 years (OR 1.15, 95% CI 1.06–1.24), immobility (OR 2.06, 95% CI 1.93–2.19), renal insufficiency (OR 2.42, 95% CI 2.15–2.71) and anemia (OR 1.75, 95% CI 1.65–1.87) predicted poor adherence to guidelines. In those with cancer, anemia (OR 1.83, 95% CI 1.64–2.06), immobility (OR 1.51, 95% CI 1.30–1.76) and metastases (OR 3.22, 95% CI 2.87–3.61) predicted long-term LMWH therapy. In conclusion, we report practices of VTE therapy in real life and found that a significant proportion of patients did not receive the recommended treatment. The perceived increased risk for bleeding has an impact on anticoagulant treatment decision.

## Introduction

For many years, the American College of Chest Physicians (ACCP) recommend that patients with acute venous thromboembolism (VTE) to be treated initially with parenteral anticoagulation (low-molecular-weight heparin [LMWH], fondaparinux or unfractionated heparin [UFH]) (Grade 1B) [1]. Then, for patients without cancer they suggest early initiation (eg, same day as parenteral therapy is started) of vitamin K antagonists (VKA) over LMWH (Grade 2C). In patients with active cancer, concordant clinical trials lead to a specific recommendation in favour of LMWH over VKA therapy since 2004 (Grade 2B) [1]. However, the implementation of recommendations on practices is the only relevant feature but little is known about patterns of management of VTE in real life, particularly after hospital discharge. Such information could possibly contribute to identify remediable gaps in patient care.

The RIETE (Registro Informatizado de Enfermedad TromboEmbólica) Registry is an ongoing, multicenter, international (Spain, Italy, France, Israel, Greece, Switzerland, Czech Republic and Macedonia), observational registry of consecutive patients with symptomatic, objectively confirmed, acute VTE. It started in Spain in 2001, and 6 years later the database was translated into English with the aim to expand the Registry to other countries, ultimately allowing physicians worldwide to use the database to select the most appropriate therapy for their patients. Data from this registry have been used to evaluate outcomes after acute VTE, such as the frequency of recurrent VTE, bleeding and mortality, and risk factors for these outcomes [2–5]. In the current analysis, we evaluated anticoagulant practices for VTE treatment over more than 10 years, and tried to identify determinants and patient-related factors for VTE management, according to international guidelines.

## Patients and Methods

Consecutive patients with symptomatic, acute deep vein thrombosis (DVT) or pulmonary embolism (PE), confirmed by objective tests (compression ultrasonography or contrast venography for DVT; helical CT-scan or ventilation-perfusion lung scintigraphy for PE), were enrolled in RIETE. Patients were excluded if they were currently participating in a therapeutic clinical trial with a blinded therapy. All patients (or their relatives) provided written or oral consent for participation in the registry, in accordance with local ethics committee requirements.

We certify that RIETE counts with IRB's approval, always in accordance with the internal requirements of each of the centers participating. This analysis was approved by the Institutional Review Board (IRB) of Hospital Universitari Germans Trias i Pujol (Badalona, Spain) and the NorthShore University HealthSystem (Evanston, Illinois, USA). As to the need for written consent, RIETE started in 2001 and then oral consent was enough. For France we got the approval of the INSERM Ethical committee for oral consent solely, as it is only an epidemiological study. However, as more and more centers have been joining us, most of them have obtained approval only if consent was written. We have the copies of these approvals.

Physicians participating in the RIETE registry ensured that eligible patients were consecutively enrolled. Data were recorded on to a computer-based case report form at each participating hospital and submitted to a centralized coordinating center through a secure website. The study coordinating center assigned patients with a unique identification number to maintain patient confidentiality and was responsible for all data management. Data quality was regularly monitored electronically, including checks to detect inconsistencies or errors, which were resolved by contacting the local coordinators. Data quality was also monitored by periodic visits to participating hospitals by contract research organizations that compared medical records with the submitted data.

### Baseline variables

The following parameters were recorded when the qualifying episode of VTE was diagnosed: patient's gender, age, and body weight and height; presence of coexisting conditions; concomitant therapies; additional risk factors for VTE including active cancer (defined as newly diagnosed cancer or cancer that is being treated [i.e. surgery, chemotherapy, radiotherapy, hormonal, support therapy, or combined treatments]) and prior VTE; laboratory data, and use of anticoagulant therapy (drug, dose, date of start and date of discontinuation for each drug).

### Treatment and Follow-up

Patients were managed according to the clinical practice of each participating hospital (i.e., there was no standardization of treatment). Patients were followed-up for up to 3 months in the outpatient clinic. During each visit, any signs or symptoms suggesting PE recurrences or bleeding complications were noted. Each episode of clinically suspected recurrent VTE was investigated by repeat CUS ultrasonography, lung scanning, helical-CT scan or pulmonary angiography as appropriate. Most outcomes were classified as reported by the clinical centers. However, if staff at the coordinating center were uncertain how to classify a reported outcome, that event was reviewed by a central adjudicating committee (less than 10% of events).

### Indicators for VTE treatment description

The ACCP Consensus Guidelines on Antithrombotic Therapy^1^ was used as a benchmark for quality of VTE care. Since 2004 and consistently in 2008 and 2012, initial treatment using heparin or LMWH with a VKA bridging at day 1 has been recommended for non-cancer-related VTE. In the same recommendations and in line with publication of major clinical trials, 3–6 months LMWH therapy has become the recommended strategy for treating patients with cancer-related VTE.

Therefore, we used the following indicators for VTE treatment description: long-term therapy was the treatment followed by the patient 7 days after the thromboembolic treatment. Patients were classified in two different strategies: long-term LMWH therapy and long-term VKA. For patients receiving long-term VKA, we separated patients starting VKA within the first week of therapy; and those receiving VKA after the first week of therapy. We reported separately patients receiving fondaparinux as initial VTE therapy.

### Statistical analysis

Every test of two proportions was a Student's t test for independent samples with a binary variable as the response (0/1). Every test of two means was a Student’s t test (if variances were homogeneous) or a Welch’s test (if variances were heterogeneous). Logistic regression models were used to examine the individual relationship between each variable and the choice of treatment in the two populations. We compared baseline characteristics of patients who were treated with: a) VKA starting within the first week; b) VKA starting beyond the first week and c) long-term LMWH therapy using chi-square tests for categorical variables (or Fisher exact test where appropriate) and ANOVA test for continuous variables. Two multivariate analyses were performed: one to identify predictors of starting VKA within the first week versus VKA starting beyond the first week or long-term LMWH in non-cancer patients; and a second analysis to identify predictors of VKA versus LMWH therapy in patients with cancer. We chose as covariates all variables with a p value <0.02 on univariate analysis and those considered by expert opinion. Logistic regression analyses were performed using a forward stepwise method with a p<0.10 and p>0.05 as inclusion and exclusion criteria using a score test for likelihood ratio criteria. All analyses were performed using a commercial software package (SPSS 20 for Mac, SPSS Inc., and Chicago, IL, USA).

## Results

Up to October 2013, 48,481 patients were recruited in RIETE. Only patients receiving VKA starting within the first 90 days after initial therapy with UFH or LMWH, or those treated with long-term therapy LMWH after an initial therapy with UFH or LMWH, were considered for the analysis. The following patients were not considered: 4,787 treated with rivaroxaban or combinations, 698 with thrombolytics, 608 undergoing an inferior vena cava filter, 26 receiving no anticoagulants and 927 experiencing an adverse event within the first 5 days. Thus, 41,625 patients were considered for the current study. Major demographic features are summarized in Table 1 for non-cancer patients and in Table 2 for those with cancer, according to choice of long-term therapy (VKA or LMWH). Long-term therapy is presented according to the country in non-cancer patients (Table 3) and in those with cancer (Table 4).

**Table 1:** Patients without cancer: Clinical characteristics according to long-term anticoagulant therapy.

		VKA start <7 days	VKA start >7 days	LMWH alone
*Patients*				
	*N*	*22*,*986*	*6*,*041*	*6*,*253*
***Clinical characteristics***				
	Gender (males)	11,313 (49%)	2,893 (48%)	2,526 (40%)^‡^
	Mean age (years±SD)	64±18	63±18^‡^	68±20^‡^
	Age >75 years	8,332 (36%)	2,015 (33%)^‡^	3,131 (50%)^‡^
	Body weight (kg±SD)	76±15	76±15	71±15^‡^
***Underlying conditions***				
	Chronic heart failure	1,473 (6.4%)	392 (6.5%)	580 (9.3%)^‡^
	Chronic lung disease	2,530 (11%)	683 (11%)	683 (11%)
	CrCl level 30–60 ml/min	7,111 (31%)	1,867 (31%)	2,477 (40%)^‡^
	CrCl levels <30 mL/min	1,098 (4.8%)	273 (4.5%)	2,105 (12%)^‡^
	Recent major bleeding	183 (0.8%)	141 (2.3%)^‡^	268 (4.3%)^‡^
	Anemia	5,248 (23%)	1,834 (30%)^‡^	2,618 (42%)^‡^
	Chronic liver disease	104 (0.5%)	33 (0.5%)	53 (0.8%)^‡^
	Inflammatory bowel disease	78 (0.3%)	34 (0.6%)*	48 (0.8%)^‡^
***Risk factors for VTE***				
	Postoperative	2,250 (9.8%)	741 (12%)^‡^	720 (12%)^‡^
	Immobility ≥4 days	4,913 (21%)	1,454 (24%)^‡^	2,502 (40%)^‡^
	Estrogen use	1,378 (6.0%)	315 (5.2%)*	177 (2.8%)^‡^
	Pregnancy/puerperium	143 (0.6%)	129 (2.1%)^‡^	315 (5.0%)^‡^
	None of the above	14,314 (62%)	3,463 (57%)^‡^	2,708 (43%)^‡^
	Prior VTE	3,875 (17%)	968 (16%)	827 (13%)^‡^
***Initial VTE presentation***				
	Pulmonary embolism	11,804 (51%)	3,212 (53%)*	2,536 (41%)^‡^
	Proximal DVT alone	9,255 (40%)	2,350 (39%)	3,094 (49%)^‡^
	Bilateral DVT alone	183 (1.6%)	80 (2.8%)^‡^	120 (3.2%)^‡^
	Upper-extremity DVT	533 (4.8%)	155 (5.5%)	245 (6.6%)^‡^

***Abbreviations*:** VKA, vitamin K antagonists; LMWH, low-molecular-weight heparin; SD, standard deviation; CrCl, creatinine clearance; VTE, venous thromboembolism; DVT, deep vein thrombosis. Differences between patients starting VKA within the first week and the other groups

* p <0.05

^‡^p <0.001.

**Table 2:** Cancer patients: Clinical characteristics according to long-term anticoagulant therapy.

		VKA start <7 days	VKA start >7 days	LMWH alone
*Patients*				
	*N*	*1*,*516*	*619*	*4*,*210*
***Clinical characteristics***				
	Gender (males)	840 (55%)	350 (57%)	2,243 (53%)
	Mean age (years±SD)	70±12	67±13^‡^	66±13^‡^
	Age >75 years	632 (42%)	199 (32%)	1176 (28%)
	Body weight (kg±SD)	74±13	73±13	71±14^‡^
***Underlying conditions***				
	Chronic heart failure	87 (5.7%)	31 (5.0%)	152 (3.6%)^‡^
	Chronic lung disease	199 (13%)	70 (11%)	377 (9.0%)^‡^
	CrCl level 30–60 ml/min	575 (38%)	226 (37%)	1,454 (35%)
	CrCl levels <30 mL/min	87 (5.7%)	30 (4.8%)	253 (6.0%)
	Recent major bleeding	15 (1.0%)	18 (2.9%)^†^	95 (2.3%)^†^
	Anemia	757 (50%)	361 (58%)^‡^	2,926 (70%)^‡^
***Cancer characteristics***				
	Metastases	455 (30%)	200 (32%)	2,550 (61%)^‡^
***Initial VTE presentation***				
	Pulmonary embolism	791 (52%)	331 (54%)	2,060 (49%)*
	Proximal DVT alone	627 (41%)	246 (40%)	1,952 (46%)^‡^
	Bilateral DVT alone	27 (3.7%)	19 (6.6%)*	143 (6.7%)^†^
	Upper-extremity DVT	46 (6.3%)	32 (11%)*	395 (18%)^‡^

***Abbreviations*:** VKA, vitamin K antagonists; LMWH, low-molecular-weight heparin; SD, standard deviation; CrCl, creatinine clearance; VTE, venous thromboembolism; DVT, deep vein thrombosis. Differences between patients staring VKA within the first week and the other groups

* p <0.05

^†^p <0.01

^‡^p <0.001.

**Table 3:** Patients without cancer: country and long-term anticoagulant therapy.

		VKA start <7 days	VKA start >7 days	LMWH alone
*Patients*				
	*N*	*22*,*986*	*6041*	*6253*
*Countries*				
	Spain	18,912 (64%)	5236 (18%)	5205 (18%)
	France	1195 (74%)	198 (12%)	227 (14%)
	Israel	424 (60%)	83 (12%)	199 (28%)
	Italy	1622 (70%)	304 (13%)	396 (17%)
	Other countries	833 (65%)	220 (17%)	226 (18%)

**Table 4:** Patients with cancer: country and long-term anticoagulant therapy.

		VKA start <7 days	VKA start >7 days	LMWH alone
*Patients*				
	*N*	*1516*	*619*	*4210*
*Countries*				
	Spain	1211 (25%)	515 (11%)	3173 (65%)
	France	63 (21%)	21 (7.0%)	215 (72%)
	Israel	29 (8.5%)	9 (2.6%)	304 (89%)
	Italy	121 (24%)	46 (9.1%)	338 (67%)
	Other countries	92 (31%)	28 (9.3%)	180 (60%)

### Patients without cancer

Among 35,280 patients without cancer, 22,986 (65%) started VKA within the first week, 6,041 (17%) beyond the first week, and 6,253 (18%) did not switch to VKA and received long-term LMWH therapy (Table 1). Overall, 65 patients received fondaparinux as initial VTE therapy: 48 (0.2%) started VKA within the first week, 11 (0.2%) beyond the first week and 6 (0.1% received long-term LMWH therapy. Compared to patients starting VKA within the first week, those starting VKA after the first week more likely to have recent bleeding, anemia, inflammatory bowel disease, recent surgery or immobilization, and more likely presented as PE initially (instead of DVT alone). Compared to those under VKA, patients receiving long-term therapy with LMWH were more likely to be female and significantly older, weighed less and more likely had chronic heart failure, renal insufficiency, recent bleeding, anemia, liver disease, chronic inflammatory disease, mental disorders (dementia, depression, schizophrenia or bipolar disorder), recent surgery, immobility or pregnancy. By contrast, they less likely had prior VTE or initial VTE presentation as PE.

### Patients with cancer

Among 6,345 patients with cancer, 1,516 (24%) started VKA within the first week, 619 (9.8%) beyond the first week, and 4,210 (66%) received long-term LMWH therapy (Table 2). Overall, 10 patients received fondaparinux as initial VTE therapy: 5 (50%) started VKA within the first week and 5 (50% received long-term LMWH therapy. Compared with patients starting VKA within the first week, those starting VKA after the first week were younger and more likely had recent surgery. Compared to those under VKA, patients receiving long-term therapy with LMWH were younger, less likely had chronic heart failure, lung disease or renal insufficiency, and more likely had anemia, immobility or metastases. They less likely had prior VTE or initial VTE presentation as PE.

### Dynamic evolution of anticoagulant prescription

**Fig 1:**
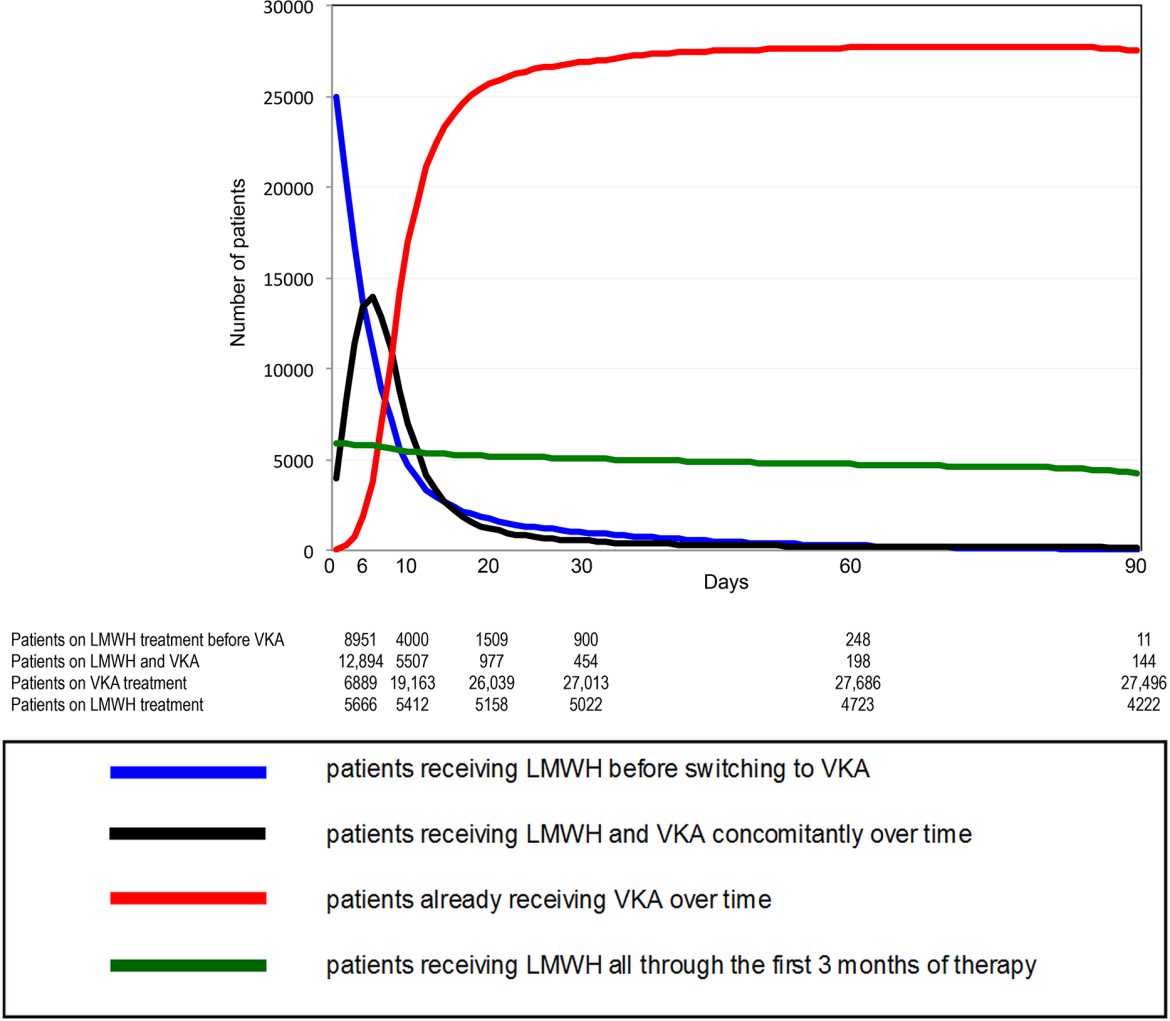
Prescription of long-term therapy within the first 90 days in non-cancer patients.

**Fig 2:**
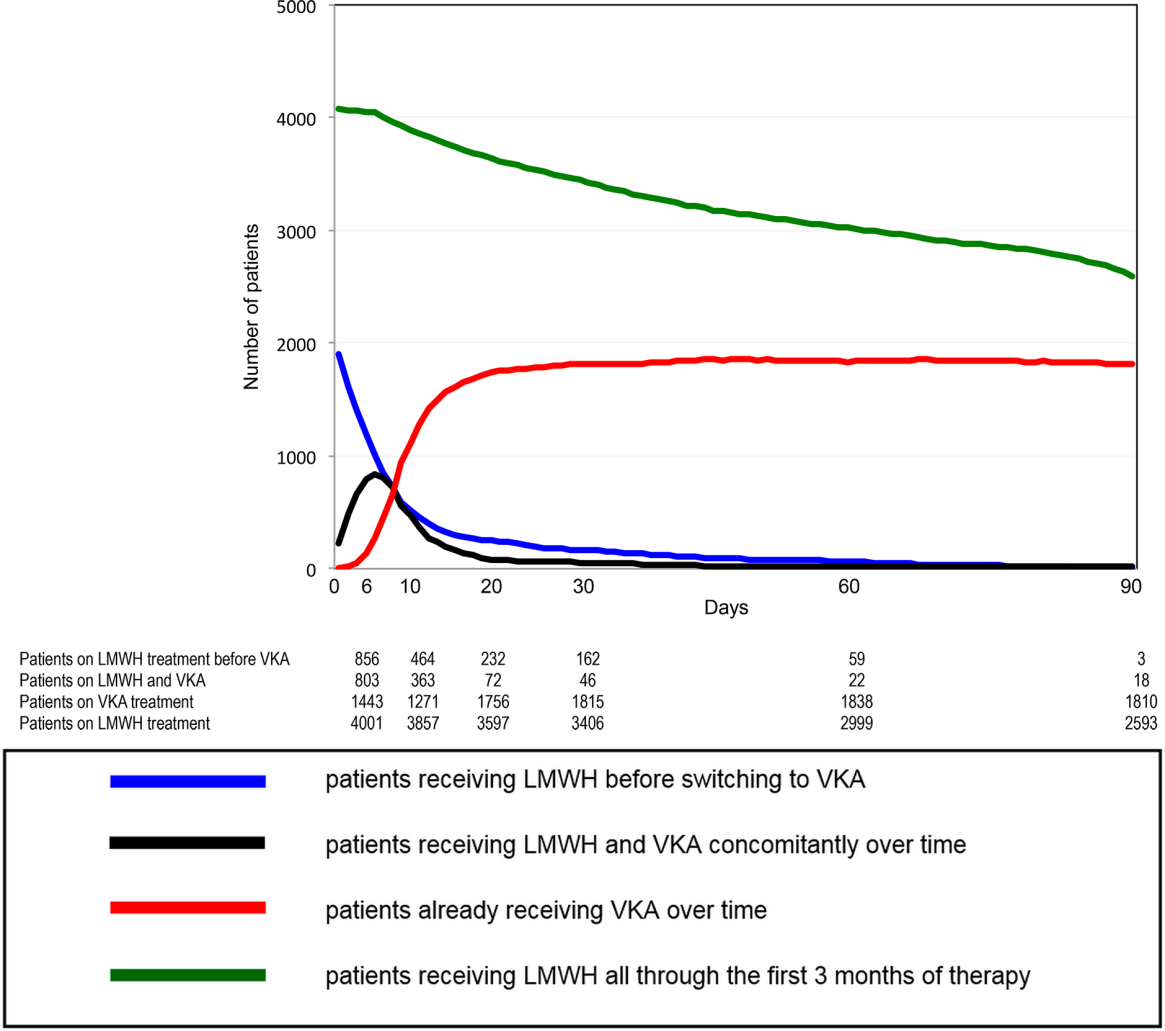
Prescription of long-term therapy within the first 90 days in patients with cancer.

**Fig 3:**
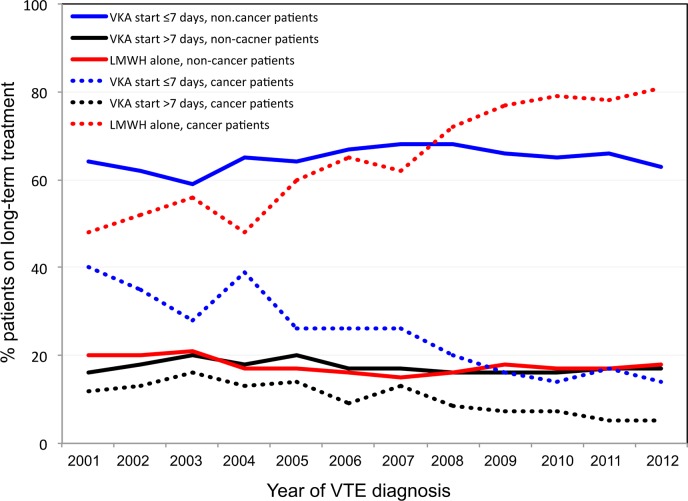
Evolution over years of prescriptions of the three modalities of long-term anticoagulant therapy in cancer and non-cancer patients.

In non-cancer patients, the number of patients receiving VKA on day 6, 10 and 90 was: 19,783, 24,670 and 26,640, respectively (Fig 1). In patients with cancer, the number of LMWH prescriptions decreased steadily over time, reflecting the poorer prognosis of patients with cancer. The amount of patients receiving VKA on day 6, 10 and 90 was: 2,246, 1,634 and 1,828 respectively (Fig 2). The evolution over time of the different anticoagulant strategies (VKA starting within the first week, VKA starting beyond the first week, long-term LMWH therapy) since the beginning of the RIETE registry is displayed in Fig 3. There was an overall stability of anticoagulant strategies in patients without cancer over time. In patients with cancer, there was a dramatic increase on long-term LMWH treatment from 2004 on, concomitantly with a decrease of VKA prescriptions.

### Factors associated with poor guidelines adherence

In patients without cancer, female gender, recent bleeding, anemia, recent surgery or immobilization, renal insufficiency, chronic liver disease, inflammatory bowel disease, mental disturbances and initial presentation as DVT independently predicted not starting VKA within the first week (Table 5). Inversely, prior VTE independently predicted a VKA initiation within the first week.

**Table 5:** Variables at baseline independently correlated to poor adherence to guidelines (VKA start <7 days for patients without cancer, and LMWH alone for patients with cancer).

		Patients without cancer	p	Patients with cancer	p
Patient characteristics		OR (95% CI)		OR (95% CI)	
	Gender (males)	0.89 (0.84–0.95)	<0.001	-	-
	Age >70 years	-	-	1.39 (1.24–1.56)	<0.001
	Chronic heart disease	-	-	1.50 (1.15–1.95)	0.003
	Chronic lung disease	-	-	1.36 (1.14–1.63)	0.001
	Recent major bleeding	2.72 (2.28–3.24)	<0.001	-	-
	Anemia	1.72 (1.62–1.83)	<0.001	0.55 (0.49–0.62)	<0.001
	Postoperative	1.20 (1.09–1.32)	<0.001	-	-
	Immobility ≥4 days	1.98 (1.86–2.10)	<0.001	0.67 (0.58–0.78)	<0.001
	Prior VTE	0.87 (0.80–0.94)	0.001	-	-
	Pulmonary embolism (vs. DVT)	0.59 (0.56–0.63)	<0.001	1.15 (1.03–1.28)	0.015
	CrCl levels >60 mL/min	1 (ref.)		-	-
	CrCl levels 30–60 mL/min	1.40 (1.30–1.52)	<0.001	-	-
	CrCl levels <30 mL/min	2.31 (2.06–2.59)	<0.001	-	-
	Chronic liver disease	1.82 (1.30–2.55)	0.001	-	-
	Inflammatory bowel disease	2.06 (1.44–2.94)	<0.001	-	-
	Mental disturbances	2.82 (2.48–3.21)	<0.001	-	-

***Abbreviations*:** OR, odds ratio; CI, confidence intervals; CrCl, creatinine clearance; VTE, venous thromboembolism; DVT, deep vein thrombosis.

In cancer patients, age >70 years, chronic heart or lung disease and initial presentation as PE independently predicted poor adherence to the ACCP recommendations (Table 3). The presence of metastases, anemia and recent immobility independently predicted the use of long-term LMWH therapy.

## Discussion

The objective of our study was to identify associations of anticoagulant use in the setting of venous thromboembolism in usual care. To achieve this objective, we obtained clinical practice-based data from the RIETE registry, the largest database on the management of symptomatic VTE. Our analyses provide useful information on clinical features of patients, management of VTE. Our data reveal that only 65% of patients without cancer started VKA within the first week, and only 66% of those with cancer received LMWH as monotherapy. Among patients without cancer, 17% started VKA therapy after the first week and 18% received long-term LMWH for at least the first 3 months.

Major factors to the anticoagulant decision were slightly different according to the presence or absence of cancer. In patients without cancer, the choice for treatment was driven by factors (recent bleeding, anemia, renal insufficiency, chronic liver disease, inflammatory bowel disease, mental disturbances) that have been associated with an increased risk for bleeding [6,7]. In patients with cancer, the choice for anticoagulation strategy was mostly influenced by the presence of metastases, anemia or immobility [8]. Of note, age and renal insufficiency influenced differently according to the presence or absence of cancer: In patients without cancer, renal insufficiency not only did not discourage clinicians from prescribing long-term LMWH, but strongly drove the decision toward long-term LMWH therapy, while age over 70 had no impact. In patients with cancer, renal insufficiency had no impact but age over 70 years discouraged clinicians from prescribing the recommended LMWH therapy. A number of studies have reported a higher incidence of major bleeding events in VTE patients receiving anticoagulant therapy in real life than in randomized clinical trials, most likely due to the strict exclusion criteria for patients recruited in these trials [9–12]. Thus, it should not be unexpected to find that the adherence to guidelines in real life was poor, as in other studies [13,14].

Interestingly, there was an evolution over time of anticoagulant strategies in patients with cancer: the publication of the CANTHANOX, CLOT and LITE studies [15–17] were associated in an increase of long-term LMWH prescriptions, as suggested in a previous study [18]. In non-cancer patients, no change has been observed over 10 years. Repeated recommendations with a high level of evidence did not result in any implementation in practice. Similar observations were made for thromboprophylaxis in medical in-patients [19,20].

Current guidelines on antithrombotic therapy provide a critical review of the literature related to the management of patients with VTE and lay the scientific groundwork for the standard of care, based largely on data from randomized controlled clinical trials [1]. However, a number of VTE patients are often excluded from randomized clinical trials. Thus, there is scarce evidence on what would be the best therapeutic approach for these patients. The RIETE Registry was designed to gather and analyze data on treatment patterns and outcomes in patients with acute VTE. In contrast to randomized controlled trials, there is no imposed experimental intervention: management is determined solely by physicians. Thus, it provides data on patients with VTE in a real-world situation with an unselected patient population. Data from RIETE are hypothesis-generating and provide feedback from real-world clinical situations. Strengths of the current analysis include that a large number of consecutive unselected patients were enrolled, and that fatal PE is by far the most important outcome during the treatment of acute PE. In our approach, we studied patients and treatment related factors contributing to anticoagulant decision. But other factors may play a role such as cost, reimbursement or need for periodical biological monitoring. Choice of treatment in patients with and without cancer is also sensitive to the individual patient’s tolerance for daily injections and perceived risk for bleeding (by the patient and the physician). Unfortunately, our registry could not assess these parameters. Of note, no country effect was observed.

### Conclusion

In conclusion, our results indicate that there are differences in practice from the guidelines, as starting VKA therapy to treat VTE in patients without cancer, and as using LMWH as monotherapy in those with cancer. These differences are likely due to comorbid conditions in patients with and without cancer and some concern about the risk of bleeding, particularly in patients who deserve quick VKA initiation. The impact of direct oral anticoagulants (which seems to have a better safety profile) may modify these unappropriated prescriptions.
